# Hypericin as a potential drug for treating Alzheimer's disease and type 2 diabetes with a view to drug repositioning

**DOI:** 10.1111/cns.14260

**Published:** 2023-05-14

**Authors:** Xin Yuan, Fei Yan, Li‐Hui Gao, Qian‐Hui Ma, Ju Wang

**Affiliations:** ^1^ School of Biomedical Engineering Tianjin Medical University Tianjin China

**Keywords:** Alzheimer's disease, drug repositioning, hypericin, type 2 diabetes

## Abstract

**Aims:**

Alzheimer's disease (AD) and type 2 diabetes (T2D) are two of the most common diseases in elderly population and they have a high rate of comorbidity. Study has revealed that T2D is a major risk factor of AD, and thus exploring therapeutic approaches that can target both diseases has drawn much interest in recent years. In this study, we tried to explore drugs that could be potentially used to prevent or treat both AD and T2D via a drug repositioning approach.

**Methods:**

We first searched the known drugs that may be effective to T2D treatment based on the network distance between the T2D‐associated genes and drugs deposited in the DrugBank database. Then, via molecular docking, we further screened these drugs by examining their interaction with islet amyloid polypeptide (IAPP) and Aβ42 peptide, the key components involved in the pathogenesis of T2D or AD. Finally, the binding between the selected drug candidates and the target proteins was verified by molecular dynamics (MD) simulation; and the potential function of the drug candidates and the corresponding targets were analyzed.

**Results:**

From multiple resources, 734 T2D‐associated genes were collected, and a list of 1109 drug candidates for T2D was obtained. We found that hypericin had the lowest binding energy and the most stable interaction with either IAPP or Aβ42 peptide. In addition, we also found that the target genes regulated by hypericin were differentially expressed in the tissues related to the two diseases.

**Conclusion:**

Our results show that hypericin may be able to bind with IAPP and Aβ42 stably and prevent their accumulation, and thus could be a promising drug candidate for treating the comorbidity of AD and T2D.

## Graphical abstract

Hypericin as the best candidate drug to inhibit the accumulation of IAPP and APP proteins and downregulate the expression of its target gene, such as GSTA1, GSTP1, NFE2L2, and CYP3A4. We provided direct evidence that hypericin can be a promising drug candidate for treating the comorbidity of AD and T2D.

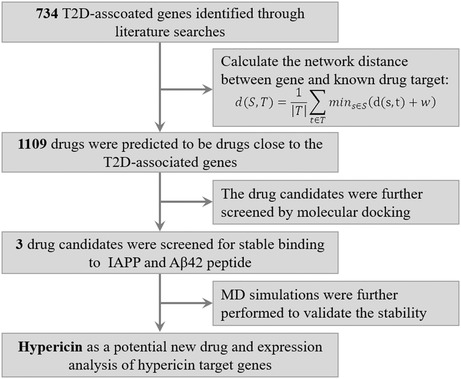

## 1 INTRODUCTION

Alzheimer's disease (AD) and diabetes are two of the most common diseases in elderly people, and their prevalence is expected to continuously increase in response to the aging population worldwide.^1^, ^2^ AD is the leading cause of dementia and it affects approximately 10% of people of 65 years or older.^3^, ^4^ Currently, there are about 50 million people with AD or other dementia globally, and the number is projected to reach about 152 million in 2050.^5^ As a metabolic disorder, diabetes affects more than 400 million patients and an estimated 193 million undiagnosed people worldwide, of which more than 90% are type 2 diabetes (T2D).^6^ Studies have shown that AD and diabetes are not independent diseases; instead, they are often closely linked at clinical and pathophysiological levels.^7^, ^8^, ^9^, ^10^ For example, diabetes is among the most common comorbidities of AD, with 25%–35% of AD patients suffering from T2D.^11^, ^12^ While in patients with diabetes, significant cognitive impairments are often observed. It is reported that T2D patients have 50%–60% or greater risk of developing AD than people without diabetes; and younger age at onset of diabetes or longer duration of the disease are usually associated with higher risk for developing AD and other dementia.^10^, ^13^, ^14^ Currently, there is still no effective treatment for AD^15^ and the failure rate of developing new drugs is very high.^16^ The high rate of comorbidity between AD and other diseases, including diabetes, brings additional difficulties and challenges to the prevention and treatment of AD.^11^

At the molecular level, the main pathological hallmarks of AD include the abnormal aggregation of amyloid‐beta (Aβ) peptides produced from sequential proteolytic cleavages of amyloid precursor protein (APP) by β‐secretase and γ‐secretase, as well as the formation of neurofibrillary tangles (NFT) formed by hyperphosphorylated tau protein in brain.^17^, ^18^ However, emerging evidence shows that insulin and insulin‐like growth factor (IGF) signaling pathways play critical roles in the cognitive function of the central nervous system (CNS) through regulating a wide range of neuronal functions, such as neuron growth, synapse formation, neurotransmission, and plasticity.^19^ An impaired insulin signaling pathway may cause metabolic and cognitive dysfunctions in the brain, and contribute to the development of AD.^20^, ^21^, ^22^ For example, hyperinsulinemia, an early sign of T2D, is associated with the increase of Aβ peptides level in the brain.^23^ Studies have also found that insulin resistance, the main characteristic of T2D, is related to the sensitivity alteration of insulin receptors in the brain of AD patients, and can further affect the expression and degradation of tau protein and Aβ peptides.^24^ In addition, it is reported that the dysregulation of β‐site amyloid precursor protein cleaving enzyme 1 (BACE1), a key gene involved in the production of Aβ in the brain, can lead to both AD‐like symptoms and the development of diabetic complications.^25^ Thus, AD is sometimes referred to as diabetes type 3.^26^, ^27^

In recent years, there is a growing interest in repositioning existing antidiabetic drugs for other diseases, and a few of them have shown great potential for treating cancer, neurodegenerative diseases, or cardiovascular diseases.^28^ Considering the close connection between AD and T2D, using medications that are already used to for diabetes to treat AD has drawn much attention.^29^, ^30^ In this regard, a few antidiabetic drugs have shown some promise.^31^, ^32^ Metformin is one of the most widely studied antidiabetic drugs in AD treatment and it is found to be associated with a reduced risk of AD in T2D patients,^33^, ^34^ or improved cognitive function in nondiabetic AD patients.^35^ Another group of antidiabetic drugs, glucagon‐like peptide‐1 (GLP‐1) receptor agonists, are shown to be able to exert neuroprotective effects in animal models of AD.^36^, ^37^, ^38^ For example, semaglutide, a GLP‐1 analog, has been extensively investigated in AD treatment due to its ability against amyloid‐β accumulation in neuron.^39^, ^40^ It can improve the motor impairment, and reduce oxidative injury, inflammation, and apoptosis in cell and animal model of AD. Progress also has been made in exploring the detailed molecular mechanisms underlying the activity of antidiabetic drug in AD treatment.^16^ For example, it is found that some genetic variants in the targets of sulphonylureas, the antidiabetic drugs that can stimulate insulin secretion by interacting with ATP‐sensitive potassium channels in the pancreas, are associated with a lower risk of AD.^41^ However, the potential neuroprotective effect of antidiabetic drugs has been challenged by other studies; for example, it is reported that long‐term use of metformin may be associated with higher risk of developing AD and other mental disorders.^42^, ^43^ Thus, it is still necessary to explore novel antidiabetic drugs that can be effective for AD treatment and elucidate their mechanism in the procedure.

In the pathological states of both T2D and AD, there is an increased production of amyloid polypeptide, which can cause the dysfunction of insulin signaling pathway, perturbations in glucose utilization, and metabolic abnormalities.^44^, ^45^ While T2D is associated with a gradual deposition of islet amyloid polypeptide (IAPP) that is coexpressed and secreted with insulin by β cells,^46^ AD is featured by an abnormal accumulation of insoluble amyloid‐β (Aβ) peptides (Aβ42) in the brain.^47^ Intriguingly, for both diseases, the deposited polypeptides have similar appearance (linear) and structure (β‐sheet), and are aggregated by self‐assembling spherical clusters of oligomers, suggesting that aberrant protein folding and aggregation are involved in both diseases.^48^ Therefore, IAPP and Aβ peptides could be potential drug targets for inhibiting Aβ self‐aggregation in both T2D and AD.

In this study, we comprehensively collected genes related to T2D, and screened the drugs from DrugBank that could be potentially used for its treatment. Then, new drug candidates were further predicted by using molecular docking and MD simulation. We identified hypericin, a naturally occurring chemical found in some species of hypericum, was able to effectively interact with both IAPP and Aβ peptides. As an inhibitor of a series of enzymes including protein kinase C (PKC), monoamine oxidase (MAO), cytochrome P450 (CYP), and dopamine β‐hydroxylase, as well as an antidepressant,^49^, ^50^ hypericin could be a potential drug candidate for treating both T2D and AD. Our work provided new insight for the prevention and treatment of AD and T2D comorbidities, as well as a theoretical basis for further development of novel therapeutic drugs.

## 2 MATERIALS AND METHODS

### 2.1 Screening novel drug candidates for T2D

A two‐step procedure was adopted to explore the potential drugs for AD and T2D. We first screened the drug candidates for T2D, then from which the molecules could target the key protein of AD was selected. Briefly, we collected the susceptibility genes related to T2D through literature searches (up to September 31, 2022) and two disease‐related databases. One database was the MalaCards (https://www.malacards.org/), which compiles disease‐associated gene‐sets derived from 73 data sources.^51^ Genes associated with T2D were queried with the default parameters. Another database was the DisGeNET (https://www.disgenet.org/), which is an integrated human disease‐associated genes and variants resource.^52^ Genes related to T2D were filtered with a threshold of gene–disease association (GDA) score >0.2, as a gene with a GDA score above 0.2 indicating a strong association with the disease. From these sources, a list of 734 unique T2D‐associated genes were retrieved.

Since these genes could be biological important in the pathogenesis of T2D, we further explored whether they could provide clues on screening potential drugs for the disease treatment. Earlier, we developed an algorithm to predict the response of a disease to drugs based on network distances between the genes associated with the disease and known drug targets.^53^ Briefly, given *S*, the set of T2D‐related genes, and *T*, the set of drug target genes and distance *d*(*s*,*t*), the shortest path length between nodes *s* (T2D‐related gene in our case; *s*∈*S*), and *t* (drug target in our case; *t*∈*T*) in the human PPI network, we have

(1) $$d\left(S,T\right)=\frac{1}{\left|T\right|}\sum_{t\in T}\min_{s\in S}\left(d\left(s,t\right)+w\right)$$

where w, the weight of a target gene, was defined as *w = −ln* (*D + 1*) if the target was one of the T2D‐related genes, and else, w = 0; *D*, the degree of a target gene in the network. In addition, a group of proteins matching the size of drug targets were randomly selected in the PPI network, then the distance d(*s*, *t*) between these simulated drug targets (representing a simulated drug) and every hub gene was calculated. The reference distribution d(*S*, *T*) represents the distance of the gene set. In this study, we calculated the distances between the hub genes and targets of drugs included in DrugBank database (https://go.drugbank.com/). A drug with smaller distance between its targets and the T2D‐associated genes is likely to be more effective to the disease. Finally, the drugs with negative distance values were selected for further analysis.

### 2.2 Molecular docking studies

The drug candidates were further screened by molecular docking. The three‐dimensional (3D) structure of IAPP protein was obtained from Protein Data Bank (PDB) (https://www.rcsb.org/),^54^ and the 3D structure of Aβ42 peptide was adopted from a previous study.^55^ Molecular docking between the screened drug candidates and IAPP or Aβ42 peptide were performed by the AutoDock Tools^56^ and the AutoDock Vina^57^ packages. Prior to molecular docking calculations, all water molecules were deleted, and then polar hydrogen and Gasteiger charges were added to the IAPP and Aβ42 peptide. Twenty rounds of docking were performed for each complex, and the protein–drug complexes with the lowest energy were kept for the subsequent analysis. The package Discovery Studio Visualizer^58^ was used to visualize the 3D and two‐dimensional (2D) structures of the binding pockets and docking results.

### 2.3 Molecular dynamics (MD) simulation and Lipinski rule of five

In addition, to evaluate the binding reliability of the compounds, MD simulations were performed. TIP3P water model were used to produce the topologies for IAPP and Aβ42 peptide. Ligand topologies were prepared with the use of ACPYPE Server.^59^ GROMACS 5.1.4 program package^60^ and the Amber 99SB‐ILDN force field^61^ was employed in MD simulation. In the setting of 310 K and 1 bar atmospheric pressure, simulation trajectories (100 ns) were analyzed in each MD system. In total, the root‐mean‐square deviation (RMSD) and root‐mean‐square fluctuation (RMSF) of the drug–protein complex were calculated by GROMACS tools. Furthermore, we also analyzed the physicochemical properties of three compounds by MolAICal (https://molaical.github.io/tutorial.html), a drug design software combined by artificial intelligence and classical programming.^62^ By this tool, the drug‐likeness information of the screened compounds, that is, the Lipinski rule of five (RO5) and PAINS (pan‐assay interference compounds), was obtained.

### 2.4 Binding free energy calculation

In order to identify the most promising drug candidate, we calculated the binding free energies of the complexes via the Molecular Mechanics Poisson–Boltzmann Surface Area (MM‐PBSA) model.^63^, ^64^ After eliminating the rotational and translational movements, MM‐PBSA has been subject to free energy calculations based on the last 20 (80–100) ns of converged MD trajectories. The binding free energies were calculated as follows:

(2) ΔGbind=ΔEvdw+ΔEele+ΔGpol+ΔGnp

where ΔGbind is the binding free energy of the complexes; and ΔEvdw, ΔEele, ΔGpol, and ΔGnp are the change in van der Waals energy, electrostatic energy, polar solvation energy, and nonpolar solvation energy upon complexation, respectively. Lastly, free energy landscapes, principal component analysis (PCA), and dynamic cross correlation matrix (DCCM) analyses were considered to analyze the dynamic behavior of protein–ligand complexes. Bio3d R package^65^ was employed to perform the residue cross‐correlation analysis.

### 2.5 Effects of hypericin on gene expression

Through our analyses, hypericin was identified as a potential drug for both T2D and AD. We further examined whether this chemical might have an effect on the expression of genes involved in the two diseases. Briefly, we collected the genes that could be regulated by this drug through DGIdb database (https://dgidb.org/)^66^ and CTD database (http://www.ctdbase.com/).^67^ Then, we went on to explore the expression of these target genes in Alzheimer's disease (http://www.alzdata.org/) and T2D (http://easybioai.com/sc2disease/home).^68^, ^69^ Genes with significant expression difference between patients and control (*p* < 0.05 and effect size of Cohen's *d* > 0.8) were selected. To better understand the functions of hypericin, we also summarized the interaction between hypericin and its target genes through literature search.

## 3 RESULTS

### 3.1 Screening novel drug candidates for AD and T2D

We first compiled a list of 734 unique T2D‐associated genes from multiple resources (Table S1). The interaction between a drug and these T2D‐associated genes would highlight the nonobvious drug–disease associations and help us to identify potential drugs that could be effective to this disease. We investigated the relationship between known drug targets and genes associated with T2D by measuring the network‐based distances between drugs and the proteins on the human interactome. By this way, drugs with shorter distances were more likely to be therapeutically beneficial than those with larger ones. Through this procedure, we assigned a distance to each of the 5595 drugs in DrugBank, from which 1109 drugs were predicted to be drugs close to the T2D‐associated genes (Table S2).

We further filtered these drugs proximal to the T2D‐associated genes to obtain the chemicals that could interact with IAPP or Aβ42 peptide, the key molecules involved in T2D or AD. Earlier studies have shown that the segment 21‐NNFGAIL‐27 is essential for the amyloidogenicity of IAPP.^54^ Notably, the region Y10‐A21 in the middle of Aβ42 peptide plays an important role in initializing the aggregation of Aβ peptides.^55^ The S‐fold in the structure of IAPP resembles polymorphs of AD‐associated Aβ fibrils, which is suggested to be related to the epidemiological link between AD and T2D.^54^ Thus, these segments could be potential binding sites for novel drug in treating the two diseases. Then, receptor–ligand interaction between IAPP or Aβ42 peptide with each of the 1109 compounds was analyzed via molecular docking (MD). The drugs were ranked by the binding energy with the segment 21‐NNFGAIL‐27 of IAPP protein and the 10 drugs with the lowest binding energy were identified as the candidates (Table 1). Importantly, all these 10 compounds could bind directly with the residue Ala21 of Aβ42 peptide and also have low binding energy.

**TABLE 1:** Candidates for drug repositioning.

Drugs	Interacting residues and binding energy (kcal/mol)		Distance to T2D‐associated genes	Status	Features of drug
	IAPP	Aβ42			
Hypericin	19, 22, 24, 26 (−7.7)	21 (−6.8)	−1.369	Investigational	An anthraquinone derivative with antidepressant, potential antiviral, antineoplastic, and immunostimulating activities
RAF‐265	15, 17, 22, 25–27 (−6.8)	21 (−7.4)	−3.742	Investigational	An orally bioavailable small molecule with potential antineoplastic activity
SLx‐4090	15, 17, 22, 25–27 (−6.8)	21 (−7.2)	−3.971	Investigational	A microsomal triglyceride transfer protein (MTTP) inhibitor potentially for the treatment of type 2 diabetes
Aplaviroc	15, 17, 25–28 (−6.7)	21 (−6.8)	−4.043	Investigational	A potent noncompetitive allosteric antagonist of the CCR5 receptor with concomitantly potent antiviral effects for HIV‐1
Efonidipine	15, 17, 22, 26, 27, 37 (−6.7)	21 (−6.6)	−1.046	Experimental	For the treatment of hypertension
Dihydroergocristine	17, 22, 23, 26 (−6.7)	10 (−7.3)	−0.092	Approved, experimental	For the treatment of cerebral and peripheric vascular events
Ciclesonide	15, 17, 19, 21, 22 (−6.6)	21 (−7.3)	−4.394	Approved, investigational	For the treatment of seasonal and perennial allergic rhinitis
Amcinonide	15, 17, 21, 22 (−6.6)	21 (−6.7)	−1.697	Approved	A corticosteroid hormone receptor agonist
5‐azaindole analog 2	15, 17, 28, 31, 37 (−6.6)	21 (−7.6)	−1.571	Experimental	Induce cell autophagy
Dihydroergocornine	17, 22, 25, 26 (−6.6)	10 (−6.9)	−0.456	Approved	Dihydroergocornine is a nootropic

**FIGURE 1:**
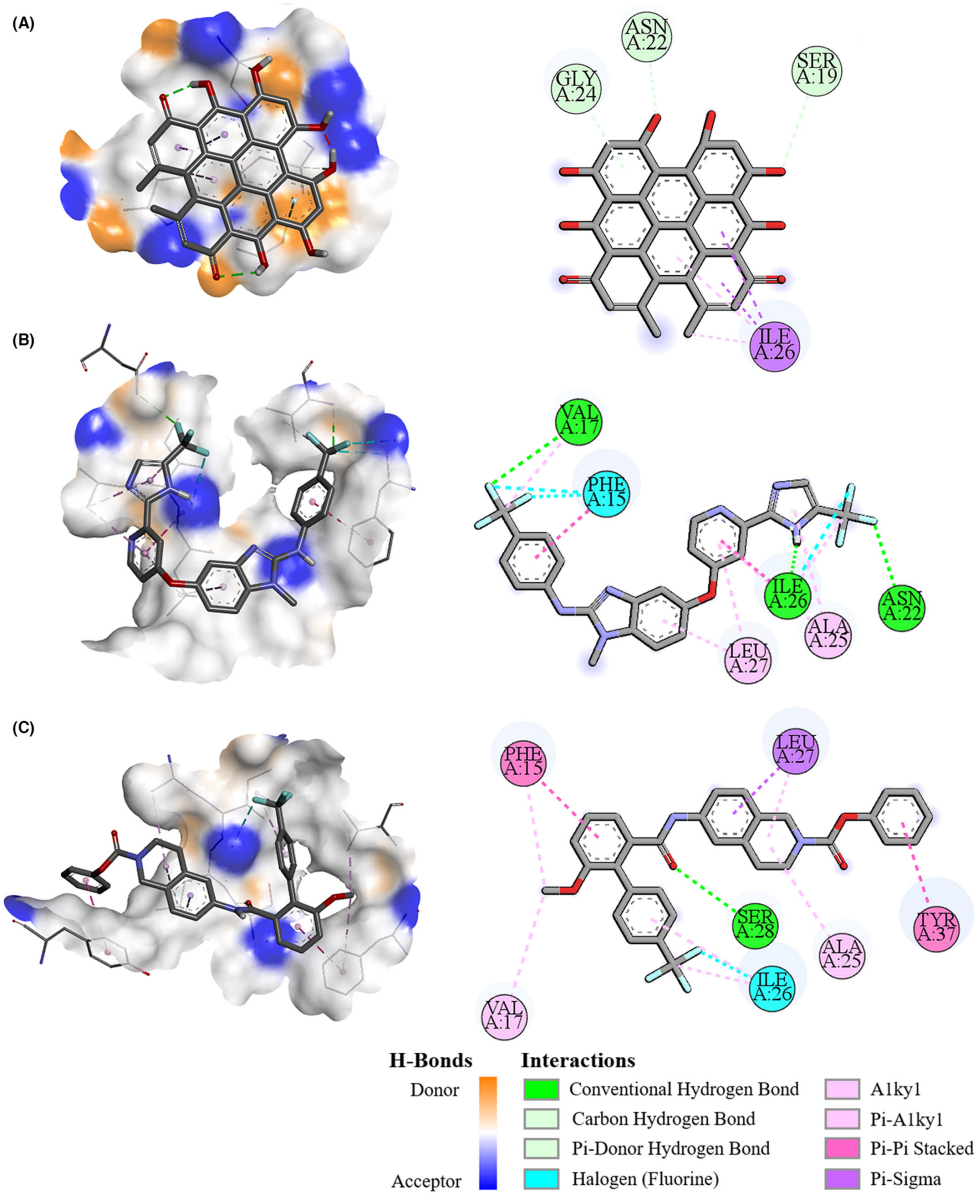
The 3D and 2D interaction diagram of IAPP protein with three compounds. Visualization of molecular docking of IAPP protein with hypericin (A), RAF‐265 (B) and SLx‐4090 binding pockets (C), respectively.

**FIGURE 2:**
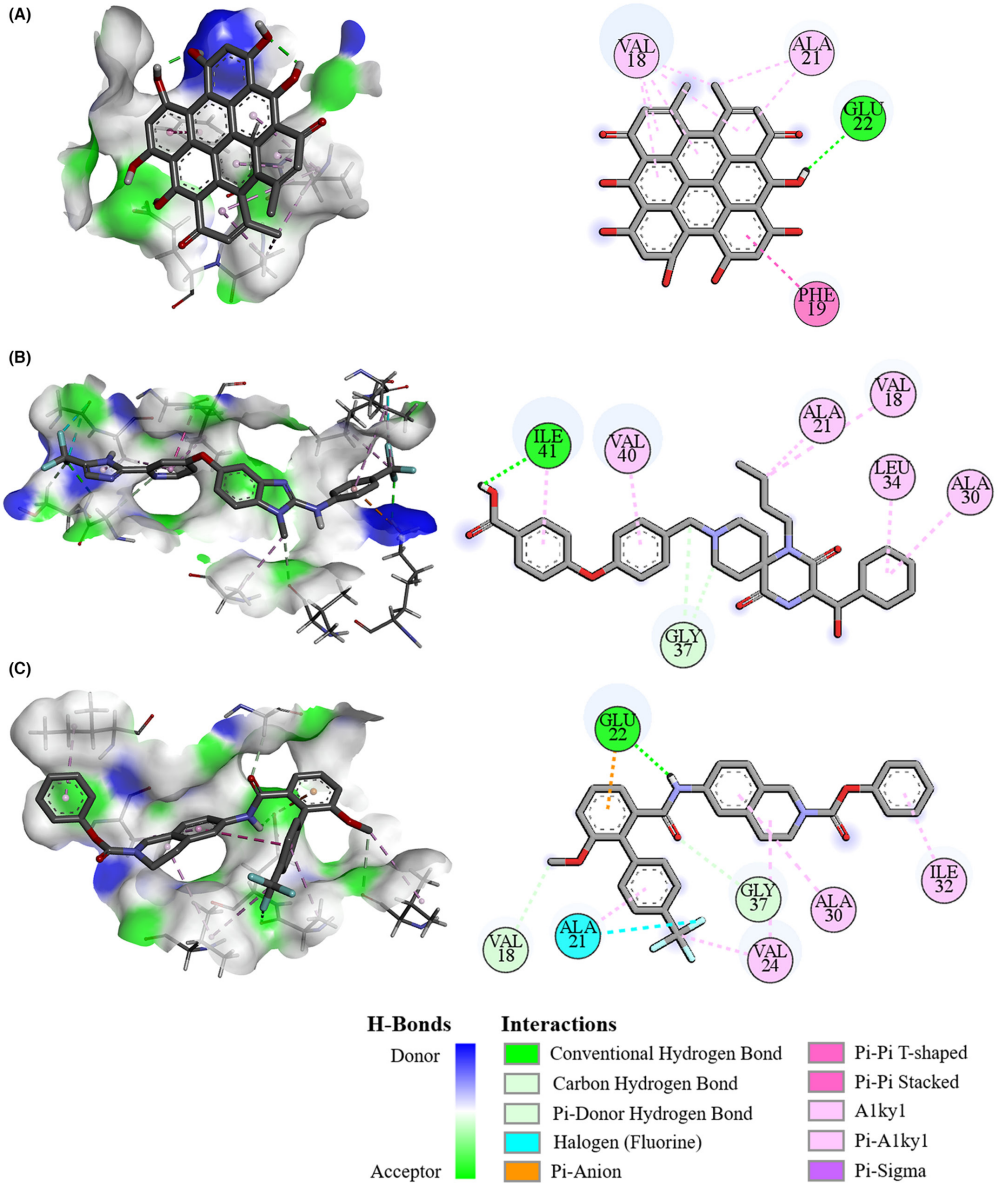
The 3D and 2D interaction diagram of Aβ42 peptide with three compounds. Visualization of molecular docking of Aβ42 peptide with hypericin (A), RAF‐265 (B) and SLx‐4090 binding pockets (C), respectively.

Then, the interaction between three of these compounds (i.e., hypericin, RAF‐265, and SLx‐4090) and IAPP or Aβ42 peptide were further explored, as each of them had relatively higher binding affinities. Some residues in the segment 21‐NNFGAIL‐27 could form hydrogen bonds with each of the three compounds, that is, Asn22 and Gly24 with hypericin (Figure 1A), Asn22 and Ile26 with RAF‐265 (Figure 1B), Ala22 and Ile26 with SLx‐4090 (Figure 1C), respectively. Unlike IAPP protein, the residue Ala21 of Aβ42 peptide could bind with these three compounds via Pi‐Alkyl and halogen interaction (Figure 2). As a result of such interaction, these three compounds may be able to inhibit the aggregation of either IAPP or Aβ42 peptide, thereby could be novel drug candidates for further study.

### 3.2 Validating the stability of three drug candidates

**FIGURE 3:**
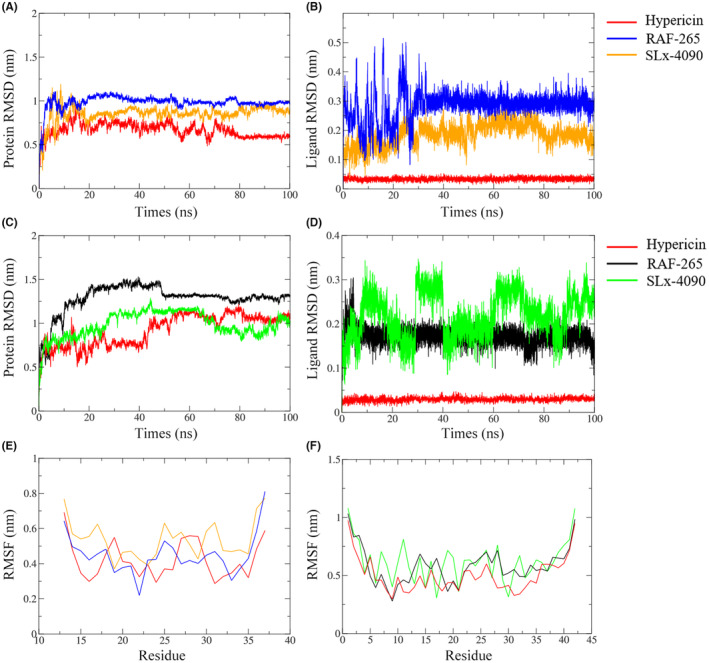
RMSD and RMSF results of the complexes systems as a function of time during the MD simulations. RMSDs of backbone for IAPP protein (A) and heavy atoms for screened three ligands (B); RMSD of backbone for Aβ42 peptide (C) and heavy atoms for screened three ligands (D); RMSF of backbone for IAPP protein (E) and Aβ42 peptide (F).

To verify the affinity of the three compounds with IAPP and Aβ42 peptide, MD simulations were further performed. We calculated RMSD and RMSF to measure the molecular stability and flexibility, a key property of biomolecules. Compared with RAF‐265 and SLx‐4090, the RMSD profiles of IAPP and ligand‐heavy atoms were the most stable in the hypericin systems during the entire simulation run (Figure 3A,B). Meanwhile, the RMSD spectrum of the hypericin complex was also found to have the most stable trajectory of Aβ42 peptide and ligand‐heavy atoms (Figure 3C,D). In addition, the RMSF of each residue was calculated for the three drug complexes. Intriguingly, they showed relatively similar fluctuations, becoming substantially less frequent in hypericin complex (Figure 3E,F). Specifically, all the trajectories tended to be equilibrated after 80 ns, with hypericin having better stabilities for IAPP and Aβ42 peptide. In addition, all the three compounds passed the PAINS test, indicating they were not false‐positive drug candidates. From the physicochemical properties point of view, hypericin exhibits better performance profile with minimum lipid–water partition coefficient (LogP) and molecular weight (MW) compared to RAF‐265 and SLx‐4090 (Table S3). Thus, hypericin seemed to be the most relevant structure for either IAPP protein or Aβ42 peptide in the small molecule drugs screened.

### 3.3 Hypericin as a potential new drug for the treatment of AD and T2D

Then, we estimated the binding energy of the protein–drug complex formed by each of the three compounds and IAPP or Aβ42 peptide by employing the MM‐PBSA method. Our results showed that hypericin (C_30_H_16_O_8_) had the strongest binding affinities with both IAPP and Aβ42 peptide (lowest binding free energy: −22.18 kcal/mol and − 35.76 kcal/mol, respectively) (Table 2), which were consistent with the fluctuation measured by RMSD and RMSF.

**TABLE 2:** Contribution of individual energy components (kcal/mol) for three drugs complexes calculated using MM‐PBSA.

Components	Hypericin		RAF‐265		SLx‐4090	
	IAPP	Aβ42	IAPP	Aβ42	IAPP	Aβ42
ΔEvdw	−29.26	−49.76	−35.84	−34.41	−30.12	−48.64
ΔEele	−1.55	−14.34	−8.90	−5.94	−8.50	−8.34
ΔGpol	12.09	32.55	27.39	20.95	23.48	33.14
ΔGnp	−2.46	−4.22	−3.46	−3.25	3.23	−5.27
ΔGbind	−22.18	−35.76	−20.81	−22.65	−18.37	−29.11

*Note:* ΔGbind (kcal/mol) represents binding affinity of protein molecule complex (Weak: −1.36 ~ −5.46; Medium: −6.83 ~ −8.19; Strong: −9.56 ~ −12.29; and Very strong: −13.66 ~ −16.39).

**FIGURE 4:**
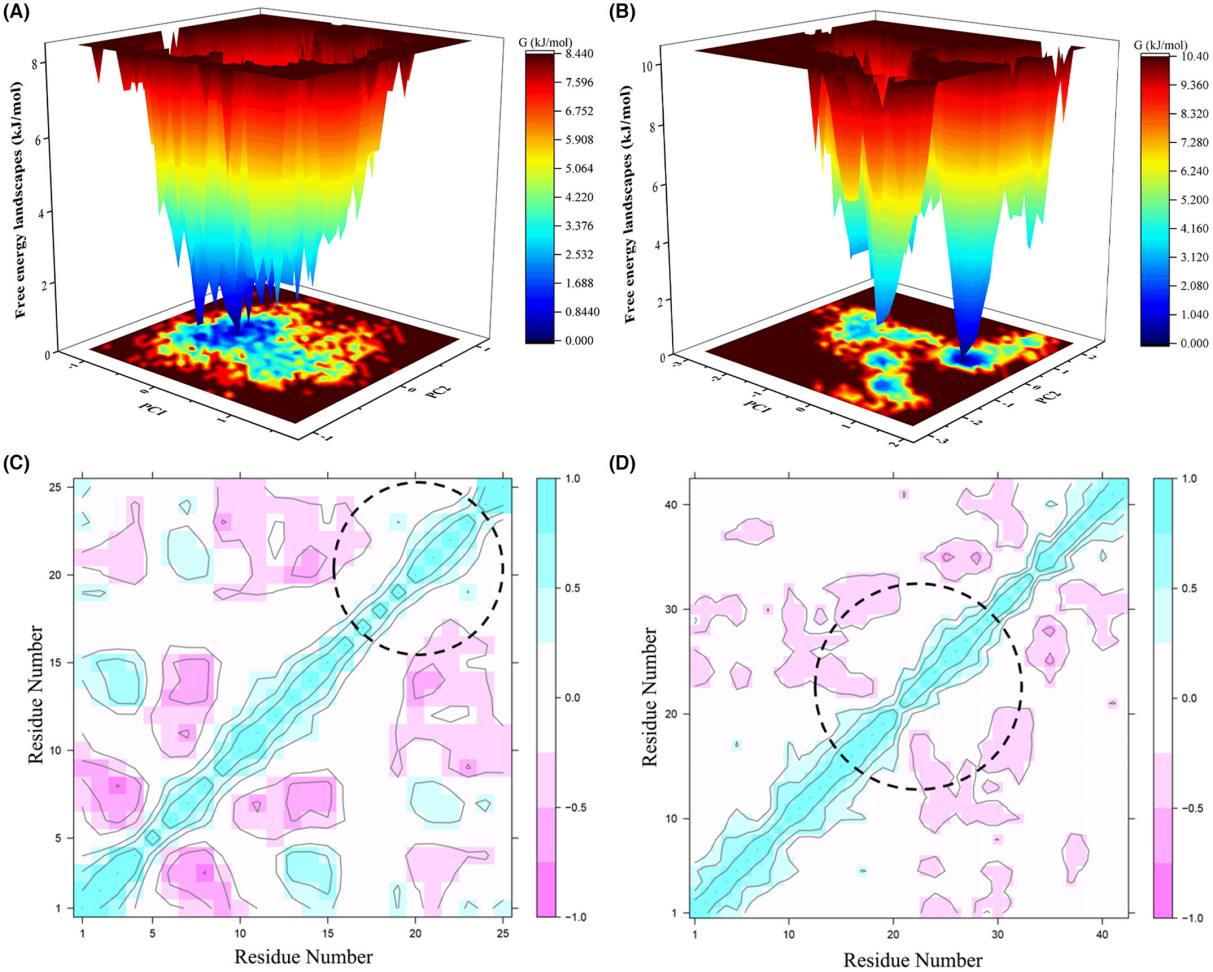
Free energy landscapes and residue correlation analysis of IAPP‐Hypericin and Aβ42‐Hypericin complex. Free energy landscapes of IAPP‐Hypericin (A) and Aβ42‐Hypericin (B); dynamic cross‐correlation matrix analysis for IAPP‐Hypericin (C) and Aβ42‐Hypericin (D). Deep blue regions in the FEL basin correspond to low energy conformations, whereas orange and deep red regions correspond to high energy or relatively unstable conformations; cyan‐colored regions of the DCCM map correspond to highly correlated motion, whereas pink‐colored regions represent highly anticorrelated motion in the protein systems.

Furthermore, the free energy landscapes corresponding to the IAPP‐hypericin and Aβ42‐hypericin complexes were generated to elucidate the conformational redistributions in protein structure caused by ligand binding events. We found that for both IAPP‐hypericin and Aβ42‐hypericin complexes, the free energy landscapes had multiple very low energy minima differentiated by small energy barriers (Figure 4A,B). The DCCM fluctuations revealed the disturbances in correlated/anticorrelated atomic fluctuations in the protein systems (Figure 4). Altogether, characterization of the distribution indicated that the segments of IAPP comprising residues 20–25 and Aβ42 peptide segment comprising residues 10–30 exhibited strongly correlated residue motions compared with other regions (Figure 4C,D). Accordingly, PCA showed a good scattering on the protein residues, which reflected an overall protein motility rate greater than 53% (Figure S1). Hence, we supposed that hypericin was able to inhibit the deposition of IAPP and Aβ42 peptide, and could be a potential drug candidate for preventing and/or treating both T2D and AD.

### 3.4 Expression analysis of hypericin target genes

To better understand the function of hypericin in biological system, we collected the genes reported to be regulated by hypericin and summarized the characteristics by checking the literature. We extracted 20 hypericin target genes from the drug–gene interaction databases (Figure S2). Of these, hypericin could directly or indirectly regulate the expression of 11 genes (GSTA1, GSTP1, ABCB1, NFE2L2, CYP3A4, MAPT, CYP1A1, CYP2D6, TOP2A, ABCC1, and ALB), whereas no evidence was found for the other nine genes (EHMT2, ALOX15B, RECQL, APEX1, THRB, POLK, BLM, KAT2A, and HPGD) (Table 3).

**TABLE 3:** Hypericin–gene interaction.

Genes	Symbol	Interaction	References
GSTA1	Glutathione S‐transferase alpha 1	Hypericin binds to GSTA1 protein and decreases its activity	^93^
GSTP1	Glutathione S‐transferase pi 1	Hypericin binds to GSTP1 protein and decreases its activity	93, 94
ABCB1	ATP binding cassette subfamily B member 1	Hypericin causes increased expression of ABCB1	95, 96
NFE2L2	NFE2 like BZIP transcription factor 2	Hypericin indirectly impairs the activation of Nrf2	^97^
ALOX15B	Arachidonate 15‐lipoxygenase type B	–	^66^
RECQL	RecQ like helicase	–	^66^
CYP3A4	Cytochrome P450 family 3 subfamily A member 4	Extract of *Hypericum perforatum* significantly decreases activity of CYP3A4 protein	^98^
APEX1	Apurinic endodeoxyribonuclease 1	–	^66^
MAPT	Microtubule‐associated protein tau	–	^66^
EHMT2	Euchromatic histone lysine methyltransferase 2	–	^66^
THRB	Thyroid hormone receptor beta	–	^66^
CYP1A1	Cytochrome P450 family 1 subfamily A member 1	Hypericin causes decreased activity of CYP1A1 protein	^99^
CYP2D6	Cytochrome P450 family 2 subfamily D member 6	Hypericin causes decreased activity of CYP2D6 protein	98, 100
TOP2A	DNA topoisomerase II alpha	Hypericin causes decreased activity of TOP2A protein	^101^
POLK	DNA polymerase kappa	–	^66^
BLM	BLM RecQ like helicase	–	^66^
KAT2A	Lysine acetyltransferase 2A	–	^66^
ABCC1	ATP binding cassette subfamily C member 1	Hypericin causes increased expression of ABCC1	95, 96, 102
ALB	Albumin	Hypericin binds to ALB protein	^93^
HPGD	15‐hydroxyprostaglandin dehydrogenase	–	^66^

**FIGURE 5:**
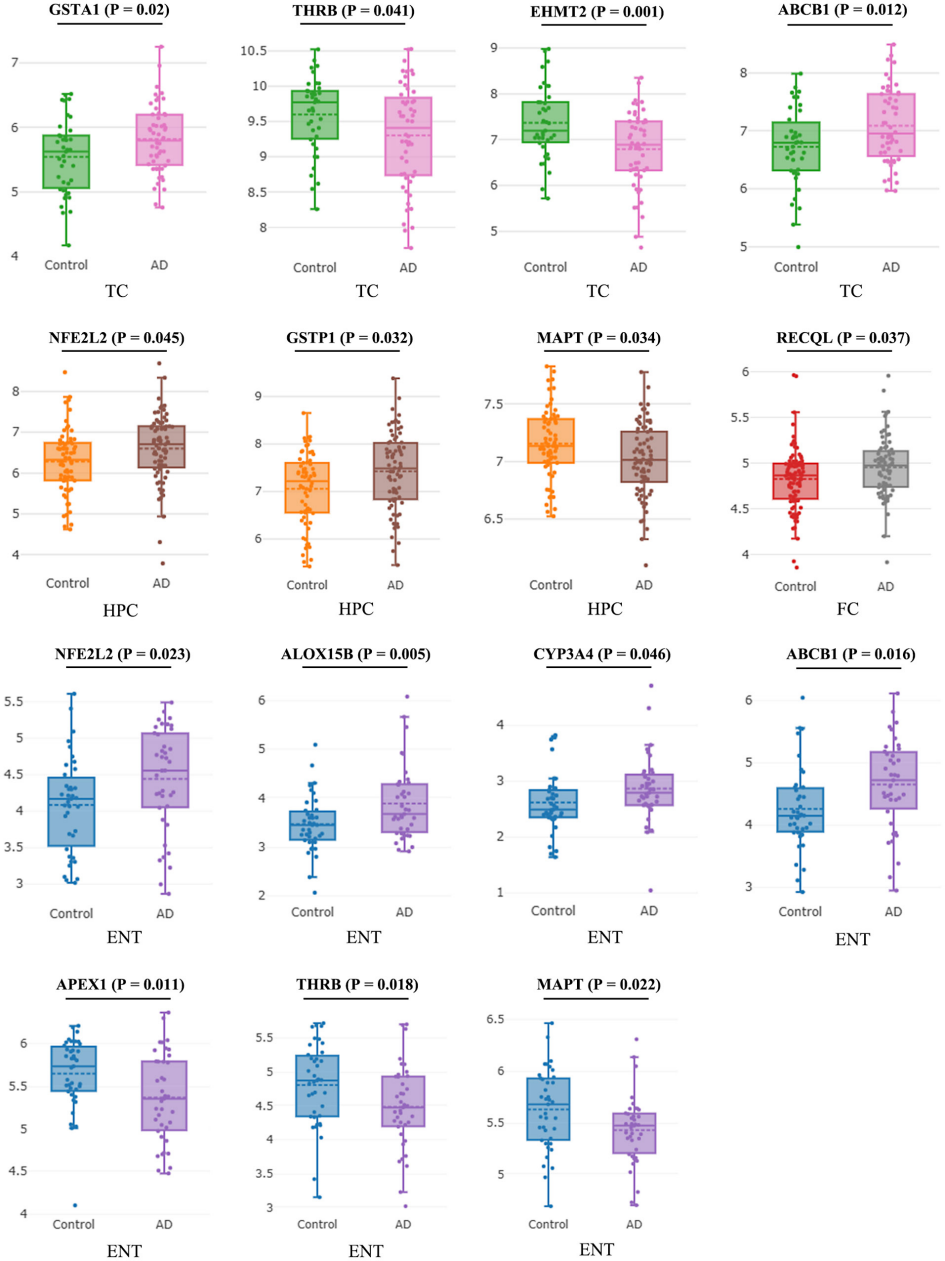
Expression of hypericin target genes in four different brain regions of normal people and AD patients. All the genes have *p* < 0.05 and effect size of Cohen's *d* > 0.8 (effect size of Cohen's *d* for genes in TC: GSTA1 = 4.700, THRB = 4.258, EHMT2 = 6.750, ABCB1 = 5.196; for genes in HPC: NFE2L2 = 4.624, GSTP1 = 4.624, MAPT = 5.366; for genes in FC: RECQL = 4.704; for genes in ENT: NFE2L2 = 4.542, ALOX15B = 5.528, CYP3A4 = 3.735, ABCB1 = 4.904, APEX1 = 5.199, and THRB = 4.593, MAPT = 4.827).

We further explored whether hypericin could regulate the expression of these 20 genes in cell lines or human brain tissues related to T2D or AD. We found that compared with the normal people, the expressions of GSTA1 and ABCB1 were upregulated, while THRB and EHMT2 were down‐regulated in the temporal cortex (TC) of AD patients; the expressions of NFE2L2 (NRF2) and GSTP1 in the hippocampus (HPC), and RECQL in the frontal cortex (FC) were upregulated, while MAPT was downregulated in the HPC; the expressions of NFE2L2, ALOX15B, CYP3A4, and ABCB1 in the entorhinal cortex (ENT) were upregulated; the expressions of APEX1, THRB, and MAPT were downregulated in the ENT (Figure 5; *p* < 0.05 and effect size of Cohen's *d* > 0.8 for all the cases). It is worth noting that GSTA1, NFE2L2, MAPT, ABCC1, APEX1, and POLK were highly expressed in T2D patients, compared to NonT2D (Figure S3, all *p* < 0.05). Thus, hypericin could regulate the expression of its target genes in the brain regions of AD patients or cells of T2D patients, providing further evidence in supporting its role in AD and T2D treatment.

## 4 DISCUSSION

Currently, there is still no effective drug for AD treatment. The available drugs can only alleviate the secondary symptoms of the disease, but fall short of reversing or delay the progression. Study has confirmed that diabetes is an important risk factor for AD, and controlling the occurrence and development of diabetes in the early stage may be a viable strategy for reducing the risk of AD.^70^ As AD and T2D are among the most common diseases in elderly population and they have a high rate of co‐occurrence, exploring drugs that can be used to treat both diseases (e.g., treating AD by antidiabetic drugs) has drawn much attention in recent years.

In this study, we screened the known drugs in DrugBank and predicted 1109 drug candidates based on the relationship of the known drugs with the T2D‐associated genes on the human interactome. Then, by analyzing the molecular interaction between the selected drugs and IAPP and Aβ42 peptide, the key proteins involved in T2D and AD, we found that hypericin had strong affinity with both peptides, indicating it could be a potential drug for treating the comorbidity of the two diseases.

Hypericin is a multifunctional agent with potential roles in antidepressive, antineoplastic, antitumor, and antiviral activity, although it is mainly used for alleviating inflammation and promoting wound healing in folk medicine.^71^, ^72^ Previous studies have also investigated the potential application of hypericin in T2D or AD treatment.^73^, ^74^ It is shown that hypericin possesses great potential as an antidiabetic drug. For example, hypericin can reduce the production of cytokines in pancreatic β‐cells, and inhibit the inflammatory process that leads to the dysfunction and death of pancreatic β‐cells.^75^ In a rat model of diabetes, hypericin is shown to have therapeutic effect on diabetic peripheral neuropathy.^76^ Also, by inhibiting islet β‐cell apoptosis and enhancing the antioxidative ability of pancreatic tissue, hypericin can alleviate β‐cell loss, and improve β‐cell mass and islet size in high‐fat/high‐sucrose (HFHS)‐induced T2D mice. In addition, hypericin treatment can improve insulin intolerance, and alleviate hyperinsulinemia.^77^ At the same time, hypericin is also a frequently used neuroactive botanical drug, and has been identified as a potential treatment against neurodegenerative diseases including AD.^78^ As we know, the aggregation of Aβ forms amyloid fibrils, which makes up the core of senile plaques, a major pathologic hallmark of AD. Currently, therapeutic intervention on the Aβ cascade, including inhibiting APP processing and Aβ production, blocking Aβ aggregation and inhibiting Aβ‐induced neurotoxicity, is among the most prominent options for AD treatment. Studies show that hypericin in low concentrations is able to partly prevent cell death induced by Aβ, and improve Aβ‐induced learning and memory impairment in mice in the Morris water maze test.^79^, ^80^ Some recent studies report that hypericin can exert neuroprotective effects, decreased NO production, and expression levels of pro‐inflammatory cytokines in hippocampal cells.^81^ Moreover, hypericin can also reverse the Aβ‐induced dysfunction in memory and increase anti‐inflammatory protein levels in mice.^82^ However, the detailed molecular mechanisms underlying the therapeutic effects of hypericin remain largely unclear.

In this study, we found that hypericin can interact with the residues 22, 24, and 26 of the IAPP fragment. After its binding, the amino acid residues, the protein showed a significant isotropic effect, suggesting a potential mechanism for the drug to inhibit IAPP aggregation. IAPP is one of the major secretory products of pancreatic β‐cells and has the ability to aggregate into insoluble amyloid fibrils that are cytotoxic to β‐cells. Due to its role in the loss and dysfunction of β‐cells, IAPP is suggested to be a therapeutic target for T2D management.^83^ The deposition of IAPP can affect a number of biological processes, such as disrupting the structure and function of cardiac myocytes and upregulating the pro‐inflammatory cytokine interleukin‐1β (IL‐1β) levels.^46^, ^84^, ^85^ Consistently, previous studies have identified some genes and pathways potentially involved in the effect of hypericin in preventing and treating diabetes. For example, hypericin can reduce cytokine‐elicited activation of both STAT‐1 and NF‐κB in a dose‐dependent manner.^75^ It is also able to mediate the activation of the cAMP/PKA/AMPK pathway caused by high‐fat‐diet‐induced metabolic abnormalities.^86^ Further, hypericin and other bioactive components in the extract from *Hypericum humifusum* may inhibit acetylcholinesterase and some key enzymes linked to T2D, or downregulate homeobox‐1 (PDX1) expression and Erk activity.^87^, ^88^ In addition, hypericin is found to be an effective inhibitor of α‐glucosidase, a major enzyme that is involved in T2D, by altering its structure and microenvironment and reducing its activity.^89^

Compared with other fragments, our results indicate that when hypericin interact with residue 21 of Aβ42 peptide, residues of the peptide show a significant isotropic effect, which may be related to the ability of the drug in inhibiting Aβ self‐aggregation. Consistently, some studies show that hypericin may regulate the formation of Aβ. For example, it is found that the extracts of *Hypericum perforatum* are able to significantly decrease the intracerebral Aβ42 levels, as well as the number and size of amyloid plaques, which can further rescue neocortical neurons and restore cognition to normal levels, and activate microglia both in vitro and in vivo.^78^ These effects are independent of hyperforin, another main component of the extracts, suggesting a potential role of hypericin.^78^, ^90^ Such results are consistent with in vitro studies showing that hypericin can interfere and significantly inhibit the formation of β‐amyloid fibrils by associating with the precursors of these fibrils.^91^ Bramant et al analyzed the secondary structure of β‐amyloid peptides in the presence and absence of hypericin, and found that hypericin can significantly interact with peptides in β‐sheet conformation and hinder the formation of fibrils.^92^

While earlier studies have clearly shown the potential of hypericin in treating T2D and AD, results from this study provide evidence that hypericin may exert its effects in AD and T2D treatment by interacting with IAPP and Aβ42 peptide to inhibit their deposition. It is noteworthy that hypericin is a small dianthrone compound (molecular mass 504 Da) with excellent stability, which could be an advantage when it is used as drug.

Further, hypericin can downregulate the expression of some of its target genes that are overexpressed in the brain of AD patients, such as GSTA1, GSTP1, NFE2L2, and CYP3A4. These genes are usually synergistic in function and are involved in the occurrence of metabolic diseases through the KEAP1‐NFE2L2 pathway and glutathione conjugation.^93^, ^94^ For this reason, GSTs and CYP450s could also be potential therapeutic targets for AD and T2D treatment. Hypericin is currently undergoing Phase I clinical trials in patients with HIV infection (NCT00000645/NCT00000792). Nevertheless, few studies have investigated the association between hypericin and AD and T2D. Existing studies have only shown that hypericin can improve symptoms, but it is unclear the mechanism underlying hypericin‐mediated. Our analysis provided evidence on its role as a potential drug for the treatment of AD and T2D at the protein and gene level.

However, there were some limitations in this study. Although several novel drug candidates including hypericin have been screened and evaluated by multiple approaches, their efficacy in AD and T2D treatment should be assessed by further experiments. Also, although hypericin is not new to the research community, our understanding on the molecular mechanisms underlying its roles in various biological activities is still incomplete. Thus, further investigation in both biochemical research and medicinal applications is necessary.

Overall, based on a drug repositioning approach, we provided direct evidence that hypericin can be a promising drug candidate for treating the comorbidity of AD and T2D. Our work will not only improve our understanding of the mechanisms or the co‐occurrence of the two diseases but also help us to explore new targets and drug candidates.

## AUTHOR CONTRIBUTIONS

XY and FY designed the experiments. XY, LG, and QM performed the data collection and analysis. XY and JW wrote the article. All authors have read and approved the final article.

## FUNDING INFORMATION

This study was supported in part by grants from National Key Research and Development Program of China (No.2016YFC0906300) and Science & Technology Development Fund of Tianjin Education Commission for Higher Education (2019KJ175).

## CONFLICT OF INTEREST STATEMENT

The authors report no conflict of interest.

## Supporting information


Figure S1

Figure S2

Figure S3

Table S1–S2

Table S3

## Data Availability

The data that supports the findings of this study are available in the supplementary material of this article.
