# Supplementary figures and images for: Hypericin as a potential drug for treating Alzheimer's disease and type 2 diabetes with a view to drug repositioning
Source: CNS Neurosci Ther. 2023 May 14;29(11):3307–21. doi: 10.1111/cns.14260 (PMC10580347; doi:10.1111/cns.14260)

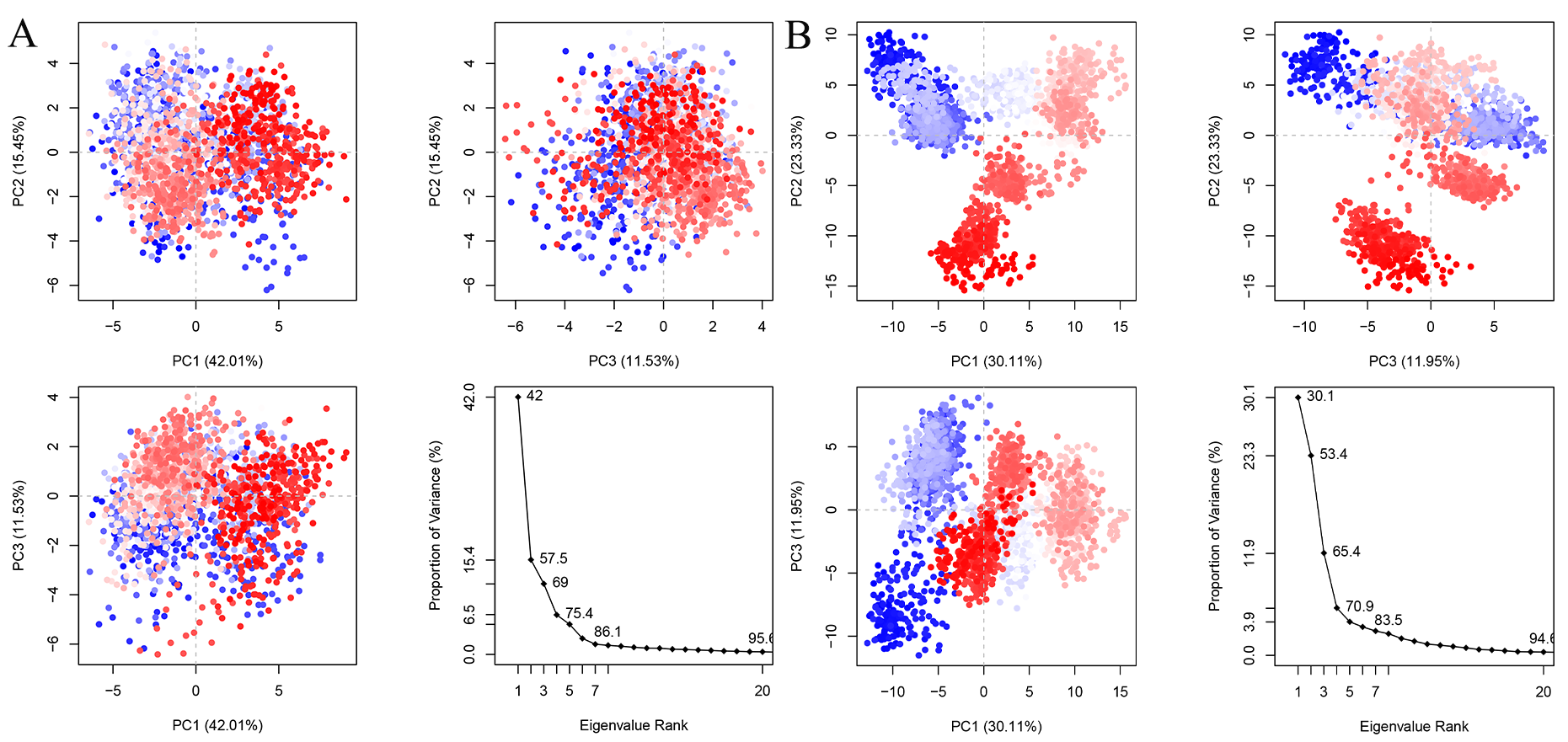

Supplement: Supplementary file 1 — Figure S1 [file CNS-29-3307-s002.tif]

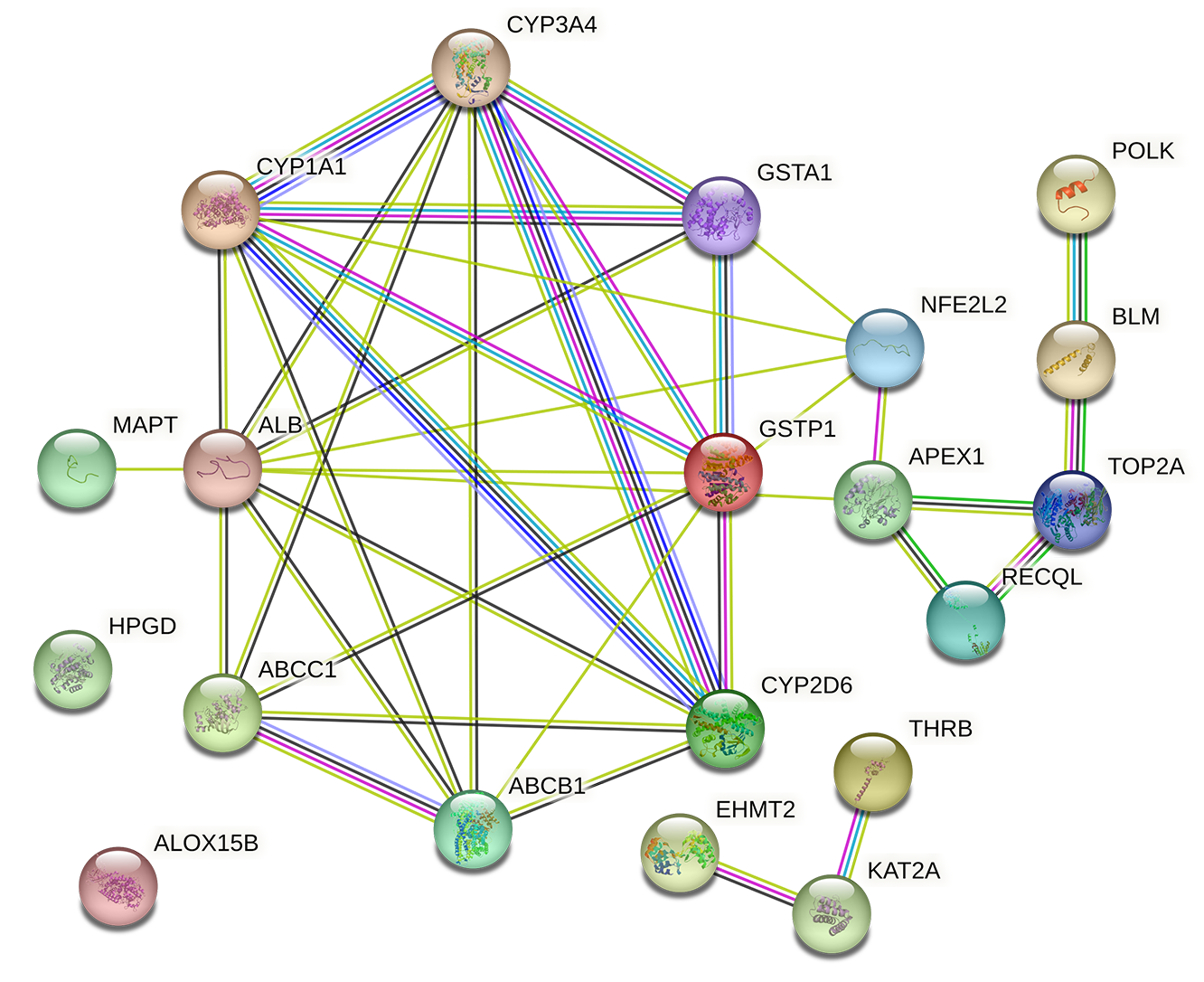

Supplement: Supplementary file 2 — Figure S2 [file CNS-29-3307-s005.zip › Supplemental Figure 2_1.tif]

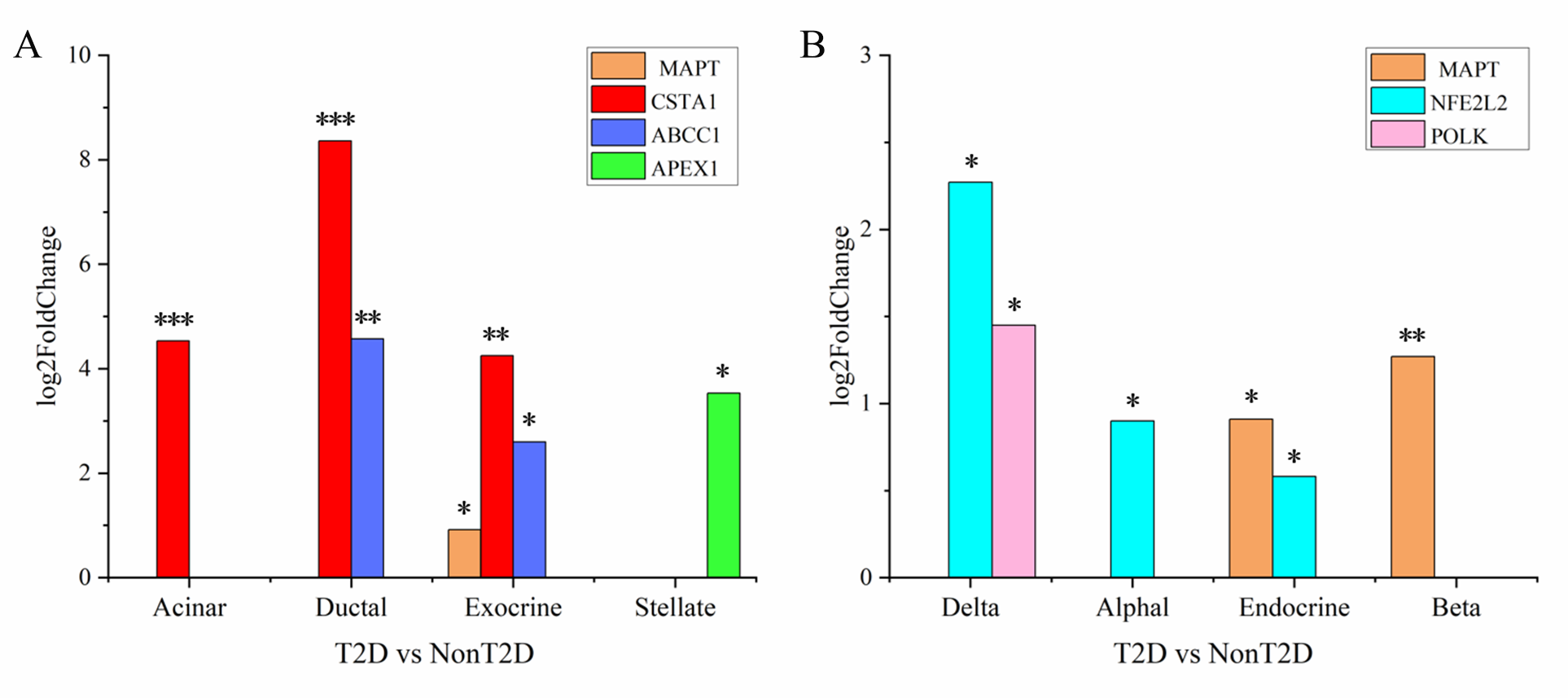

Supplement: Supplementary file 3 — Figure S3 [file CNS-29-3307-s004.tif]
