# Supplementary material for: Hypericin as a potential drug for treating Alzheimer's disease and type 2 diabetes with a view to drug repositioning
Source: CNS Neurosci Ther. 2023 May 14;29(11):3307–21. doi: 10.1111/cns.14260 (PMC10580347; doi:10.1111/cns.14260)
Supplement: Supplementary file 5 — Table S3 [file CNS-29-3307-s003.doc]

Supplementary Table S3. The physicochemical and pharmacokinetic properties typical for three small-molecule drugs.

| Drugs | LogP | HBD | HBA | MW | Rotatable bonds | PAINS or not |
| --- | --- | --- | --- | --- | --- | --- |
| Hypericin | 1.740 | 6 | 2 | 504 | 0 | No |
| RAF-265 | 6.783 | 2 | 1 | 518 | 7 | No |
| SLx-4090 | 8.593 | 1 | 0 | 546 | 7 | No |

Note: LogP, lipid-water partition coefficient; HBD, hydrogen bond donor; HBA, hydrogen bond acceptor; MW, molecular weight; PAINS, pan-assay interference compounds.
